# Deaths Associated with Pneumonic Plague, 1946–2017

**DOI:** 10.3201/eid2610.191270

**Published:** 2020-10

**Authors:** Alex P. Salam, Amanda Rojek, Erhui Cai, Mihaja Raberahona, Peter Horby

**Affiliations:** United Kingdom Public Health Rapid Support Team, London, UK (A.P. Salam);; Centre for Tropical Medicine and Global Health, University of Oxford, Oxford, UK (A.P. Salam, E. Cai, M. Raberahona, P. Horby);; Centre Hospitalier Befelatanana, Antananarivo, Madagascar (M. Raberahona);; Centre for Integrated Critical Care, University of Melbourne, Melbourne, Victoria, Australia (M. Raberahona)

**Keywords:** plague, *Yersina pestis*, outbreak, pneumonia, pneumonic, mortality, bacteria, Madagascar, vector-borne infections, United Kingdom, Australia

*Yersinia pestis*, the causative agent of plague, is a Tier 1 select agent because of the high case-fatality rate associated with pneumonic plague and its potential as a bioterrorism agent in aerosolized form (https://emergency.cdc.gov/agent/agentlist-category.asp). The death rate for persons with untreated primary pneumonic plague was reported to be almost 100% (*1*); the death rate for persons treated for primary pneumonic plague was 50% (*1*). Overall, the death rate for persons treated for primary pneumonic plague was high despite the sensitivity of *Y. pestis* to aminoglycosides, quinolones, and tetracyclines (*2*,*3*) and the relatively good penetration of some of these antimicrobial drugs into lungs (*4*,*5*). During the 2017 Madagascar pneumonic plague outbreak, the observed death rate for treated persons appeared to be substantially lower than that reported in the literature (*6*). Many articles that quoted a 50% death rate for treated primary pneumonic plague were cited in a 2000 study by Ratsitorahina et al. (*7*), which described a small outbreak in Madagascar in 1997. The article indicated that the data showed an overall death rate of 53% but did not state the number of deaths. However, the death rate for treated persons with confirmed or probable plague was 10%. On reviewing reports that cited Ratsitorahina et al., we identified 9 studies that referenced 50% of persons treated for pneumonic plague who died, 1 study that referenced 40%, and none referencing lower rates. One was a review cited 9 times about persons treated for primary pneumonic plague for whom the death rate was 50%. We identified 6 reports that stated but did not reference a 50% death rate for persons treated for pneumonic plague.

## The Study

To address the lack of evidence supporting the frequently cited 50% death rate for persons treated for primary pneumonic plague, we conducted a systematic review and meta-analysis. We followed PRISMA (Preferred Reporting Items for Systematic Reviews and Meta-Analyses, http://www.prisma-statement.org) and MOOSE (Meta-analysis of Observational Studies in Epidemiology [*8*]) guidelines. The study was prospectively registered on PROSPERO (CRD42018086223) (https://www.crd.york.ac.uk/PROSPERO).

We searched PubMed and Embase covering 1946–2017 using the search terms “Yersinia pestis” or “plague” and “pneumon*” and limited our search to human data. We searched references and included articles describing death (within a 28-day period from illness onset) among patients with confirmed, probable, and suspected primary or undifferentiated (i.e., primary or secondary not distinguished pneumonic plague 1999 World Health Organization case definition, https://www.who.int/csr/resources/publications/plague/WHO_CDS_CSR_EDC_99_2_EN/en/). We did not restrict by study type, language, or minimum patient number.

Two authors reviewed and extracted data; a third author resolved any disagreements. Data fields extracted included year and country of the outbreak, number of patients who survived and died (stratified by antimicrobial drug status), number of patients receiving different antimicrobial drug classes, time to antimicrobial drug administration, and receipt of plague vaccination or prophylaxis (these patients were excluded). We calculated the risk from the number of events and participants in each group.

We performed a meta-analysis using a binomial-specific method. We assessed heterogeneity using the χ^2^ test and quantified results with the *I*^2^ statistic. In addition, we preplanned 2 sensitivity analyses to examine whether our estimation of death was influenced by the inclusion of specific articles (pneumonic plague was not confirmed as primary disease or patients with suspected and probable disease). We conducted statistical analysis using R version 3.6.0 (R Project, https://www.r-project.org).

We reviewed 362 articles (Appendix Figure 1). We described 1,107 patients in 44 articles (Appendix Table). Twenty-nine articles reported antimicrobial drug use in 108 patients with confirmed or probable pneumonic plague. For pneumonic plague patients receiving antimicrobial drug therapy, the pooled death rate was 17% (95% CI 8%–31%; *I*^2^ = 47%) (Appendix Figure 2). Pneumonic plague patients who did not receive antimicrobial drug therapy had a pooled death rate of 98% (95% CI 73%–100%; I^2^ = 47%) (Appendix Figure 3). Pneumonic plague patients for whom antimicrobial drug status was unknown had a pooled death rate of 46% (95% CI 32%–61%) (Appendix Figure 4). Heterogeneity was significant (*I*^2^ = 91%; p<0.01). The pooled death rates were similar when sensitivity analysis was conducted (Table). Antimicrobial drugs in the reports were aminoglycosides (90 courses), quinolones (24 courses), sulfonamides (22 courses), chloramphenicol (16 courses), tetracyclines (14 courses), and cotrimoxazole (3 courses). Six reports described time to from admission to antimicrobial drug administration, but the nonstandardized reporting precluded stratification by this measure.

**Table:** Sensitivity analysis of antimicrobial drug use and rates of pneumonic plague–related deaths, 1946–2017

Antimicrobial drug use	Confirmed cases, % (95% CI)	Total cases, % (95% CI)
Treated		
Primary plague	27 (14–47)	6 (1–31)
Undifferentiated	28 (6–72)	6 (1–31)
Not treated		
Primary plague	94 (82–98)	99 (22–100)
Undifferentiated	No data	100*
Unknown		
Primary plague	No data	29 (13–51)
Undifferentiated	42 (23–64)	51 (31–71)

*Crude analysis; model fails under this condition.

## Conclusions

Our meta-analysis identified a 17% death rate for persons treated for pneumonic plague, in contrast to the 50% often reported in the literature. The death rate for the 2017 Madagascar outbreak was published after we completed our systematic review but is consistent with our findings (25% in confirmed cases) (*9*). These figures compare with 13.6% for patients who died in the hospital of community-acquired pneumonia; 12.3% who died of *Streptococcus pneumoniae* infection; 14.7% who died of *Legionella* species infection; and 32%–61% who died of *Staphylococcus aureus*, *Escherichia coli*, *Klebsiella* species, or *Pseudomonas aeruginosa* infections (*10*). However, persons who died of other etiologic causes were predominantly elderly and had underlying conditions (*10*).

Our review indicated insufficient standardized data to stratify death by time from symptom onset to antimicrobial drug administration. The literature we assessed often stated that pneumonic plague is fatal in almost all patients who start antimicrobial drugs >24 hours after symptom onset. Generally, descriptions cite either 1 article, in which 11 patients treated within 24 hours survived and 2 treated after 24 hours died (*11*), or a handful of isolated case reports. However, case reports and series also exist in which patients survived despite starting antimicrobial drugs >24 hours after symptom onset (*12*–*14*).

An accurate estimate of death is crucial for several reasons. First, it is helpful for public health planning during outbreaks, including the allocation of healthcare resources and the development of social mobilization campaigns. The commonly reported high death rate associated with primary pneumonic plague contributes to fear and panic among healthcare workers and the public. For example, anecdotal reports indicating concerns during the Madagascar outbreak were the following: healthcare workers taking continuous antimicrobial drug prophylaxis, mass public use of over-the-counter antimicrobial drugs, asymptomatic persons visiting the hospital, and sick persons avoiding the hospital. Accurate assessment of death is also essential for clinical trial design. For example, the required sample size would be 134 (power 0.8, α = 0.025) for a binary outcome superiority trial in which the death rate in the control arm was 50% and the intervention was assumed to reduce death by 50% (similar to the assumptions in a clinical trial of gentamicin vs. doxycycline in Tanzania in 2002) (*15*). However, a sample size of 476 would be required in a trial in which the death rate in the control arm was 20%. A sample size renders a superiority trial unfeasible. Even during the Madagascar outbreak, the largest outbreak of pneumonic plague this century, the final number of confirmed pneumonic plague patients was only 32 (*9*).

The major limitation of our meta-analysis is the sporadic reporting of clinical data and the relatively small number of cases for which antimicrobial drugs treatment status was described. Reporting bias in the literature also is likely, and pneumonic plague patients who survive are more likely than those who do not to be reported. Nonetheless, data indicate that the percentage of persons who die of treated pneumonic plague appears to be substantially lower than is frequently reported in the literature.

Appendix Additional information in a meta-analysis of deaths associated with pneumonic plague, 1946–2017.
