# Supplementary material for: Deaths Associated with Pneumonic Plague, 1946–2017
Source: Emerg Infect Dis. 2020 Oct;26(10):2432–4. doi: 10.3201/eid2610.191270 (PMC7510718; doi:10.3201/eid2610.191270)
Supplement: Appendix — Additional information in a meta-analysis of deaths associated with pneumonic plague, 1946–2017. [file 19-1270-Techapp-s1.pdf]

Article DOI: https://doi.org/10.3201/eid2610.191270

Deaths Associated with Pneumonic Plague, 1946–2017

Appendix

Articles Citing a 50% Mortality in Pneumonic Plague

Many articles that quote a treated mortality of 50% cite a 2000 Lancet paper by Ratsitorahina et al. (*1*), which describes a small outbreak of primary pneumonic plague in Madagascar in 1997. The mortality is not explicitly stated in the article, but the data show an overall mortality of 50%. However, the mortality amongst treated confirmed/probable cases is reported as only 10%. On reviewing publications that cited Ratsitorahina et al., we identified 9 that referenced a treated mortality of 50%, one of 40%, and none referencing other values (*2–11*). None of the articles explained how they derived this figure. One of these citations was a review paper published in the Lancet in 2007 (*11*). This review paper was also itself cited 9 times in relation to a treated mortality of 50% (*12–19*). Through examination of these publications' references, we identified a further 6 publications where a treated mortality of 50% was stated but not referenced (*20–25*), and only one publication mentioning a lower mortality (14%; source unreferenced) (*26*).

In the Ratsitorahina et al. paper, there were 18 cases in total. One patient (case 10) did not have plague. Therefore, there were 17 cases with plague. Of these, 10 appear to have received treatment (cases 6, 7, 8, 9, 11, 12, 13, 14, 15, 16 – as "eight patients with pneumonic syndrome were transferred" . . . "The patients were suspected to have pneumonic plague and thus were treated with streptomycin," and "The healer's brother died a few hours after admission"). Only 1 died out of these 10 (case 6). Eight patients did not receive treatment, and all died (cases 1, 2, 3, 4, 5, 17, 18). Therefore, the treated mortality is 10%, the untreated mortality is 100%, and the overall mortality is 53%.

Methods

We detailed whether confirmed cases alone, confirmed and probable cases together, or confirmed, probable, and suspected cases together were reported (and extracted numerical

data on the most specific classification available). Likewise, we noted whether articles included only primary pneumonic or undifferentiated pneumonic cases (defined by article authors using the term ‘primary’) and extracted data on the most specific measurement. Data on secondary pneumonic cases were not included. We defined antibiotic treatment as one or more doses of antibiotics currently or previously used for the treatment of plague: aminoglycosides, quinolones, tetracyclines, sulphonamides (alone), septrin, and chloramphenicol. Where data were duplicated in more than one publication, the largest of the overlapping cohorts that detailed antibiotic use were used. Data were reported as missing for each variable when they were not available in the article. Authors were not contacted for missing data. Where data were duplicated in more than one publication, the largest of the overlapping cohorts that detailed antibiotic use were used.

Descriptive statistics are presented as frequencies for categorical variables, means and standard deviations for normally distributed data, and median with range for other continuous variables.

Results

Antibiotics given included aminoglycosides (90 courses), quinolones (24 courses), sulphonamides (23 courses), chloramphenicol (16 courses), tetracyclines (15 courses), and septrin (3 courses). Six articles described the time to antibiotic administration in some detail, but the non-standardised way this was reported precluded stratification by this measure.

References

1. Ratsitorahina M, Chanteau S, Rahalison L, Ratsifasoamanana L, Boisier P. Epidemiological and diagnostic aspects of the outbreak of pneumonic plague in Madagascar. Lancet. 2000;355:111–3. PubMed https://doi.org/10.1016/S0140-6736(99)05163-6

2. Pechous RD, Sivaraman V, Stasulli NM, Goldman WE. Pneumonic plague: the darker side of *Yersinia pestis.* Trends Microbiol. 2016;24:190–7. PubMed https://doi.org/10.1016/j.tim.2015.11.008

3. Sexton DJ, Calderwood S, Bloom A. Clinical manifestations, diagnosis, and treatment of plague (*Yersinia pestis* infection). UpToDate [cited 2018 Dec 6]. https://www.uptodate.com/contents/clinical-manifestations-diagnosis-and-treatment-of-plague-yersinia-pestis-infection

4. Amedei A, Niccolai E, Marino L, D'Elios MM. Role of immune response in *Yersinia pestis* infection. J Infect Dev Ctries. 2011;5:628–39. PubMed https://doi.org/10.3855/jidc.1999

5. El-Bahnasawy MM, Gabr MSA, Abdel-Fattah MA, Gaber WAI, Morsy TA. Is plague a problem in the Egyptians returning back from Libya? J Egypt Soc Parasitol. 2012;42:329–48. PubMed https://doi.org/10.12816/0006321

6. Dunnick CA. Life-threatening dermatoses and emergencies in dermatology. JAMA. 2010;303:1756. https://doi.org/10.1001/jama.2010.561

7. Martínez-Chavarría LC. *Yersinia pestis*-host immune cells interactions at early events during bubonic plague infection. Current Tropical Medicine Reports [cited 2016 Apr 6]. https://link.springer.com/article/10.1007/s40475-016-0071-5

8. Jahanian-Najafabadi A, Soleimani M, Azadmanesh K, Mostafavi E, Majidzadeh-A K. Molecualr Cloning of the capsular antigen F1 of *Yersinia pestis* in pBAD/gIII plasmid. Res Pharm Sci. 2015;10:84–9. PubMed

9. Mwengee W, Butler T, Mgema S, Mhina G, Almasi Y, Bradley C, et al. Treatment of plague with gentamicin or doxycycline in a randomized clinical trial in Tanzania. Clin Infect Dis. 2006;42:614–21. PubMed https://doi.org/10.1086/500137

10. Choi H. *Salmonella* suppress innate immunity by targeting mast cells [dissertation]. Durham (NC): Duke University; 2014.

11. Prentice MB, Rahalison L. Plague. Lancet. 2007;369:1196–207. PubMed https://doi.org/10.1016/S0140-6736(07)60566-2

12. Lister IM, Mecsas J, Levy SB. Effect of MarA-like proteins on antibiotic resistance and virulence in *Yersinia pestis.* Infect Immun. 2010;78:364–71. PubMed https://doi.org/10.1128/IAI.00904-09

13. Barros, M. Caracterização Genética de cepas de *Yersinia pestis*. Dissertaçoes de Mestrado-Genética. 2012;22:1–145.

14. Cao L, Lim T, Jun S, Thornburg T, Avci R, Yang X. Vulnerabilities in *Yersinia pestis* caf operon are unveiled by a *Salmonella* vector. PLoS One. 2012;7:e36283. PubMed https://doi.org/10.1371/journal.pone.0036283

15. Aubron C. Critical factors for parameterisation of disease diagnosis modelling for anthrax, plague and smallpox. Australia: Human Protection and Performance Division and Defence Science and Technology Organisation; 2012.

16. Alvarez ML, Cardineau GA. Prevention of bubonic and pneumonic plague using plant-derived vaccines. Biotechnol Adv. 2010;28:184–96. PubMed https://doi.org/10.1016/j.biotechadv.2009.11.006

17. Foster CL, Mould K, Reynolds P, Simonian PL, Erlandson KM. Clinical problem-solving. Sick as a dog. N Engl J Med. 2015;372:1845–50. PubMed https://doi.org/10.1056/NEJMcps1411346

18. van Lier CJ, Sha J, Kirtley ML, Cao A, Tiner BL, Erova TE, et al. Deletion of Braun lipoprotein and plasminogen-activating protease-encoding genes attenuates *Yersinia pestis* in mouse models of bubonic and pneumonic plague. Infect Immun. 2014;82:2485–503. PubMed https://doi.org/10.1128/IAI.01595-13

19. Endom E. Bioterrorism and the pediatric patient: an update. Clin Pediatr Emerg Med. 2013;14:102–17. https://doi.org/10.1016/j.cpem.2013.04.001

20. Donaires LF, Céspedes M, Valencia P, Salas JC, Luna ME, Castañeda A, et al. Primary pneumonic plague with nosocomial transmission in La Libertad, Peru 2010 [in Spanish]. Rev Peru Med Exp Salud Publica. 2010;27:326–36. PubMed https://doi.org/10.1590/S1726-46342010000300004

21. Gradon JD. Plague Pneumonia. Curr Infect Dis Rep. 2002;4:244–8. PubMed https://doi.org/10.1007/s11908-002-0087-y

22. de Almeida AMP, Leal NC, editors. Advances in *Yersinia* research. New York: Springer Science & Business Media; 2012.

23. Boire NA. Lessons learned from historic plague epidemics: the relevance of an ancient disease in modern times. Journal of Infectious Diseases and Preventative Medicine. 2014;02:1–18. https://doi.org/10.4172/2329-8731.1000114

24. Schriefer ME. *Yersinia*. Manual of clinical microbiology. 11th ed. Washington (DC): American Society for Microbiology Press; 2015.

25. Marcondes CB, editor. Arthropod borne diseases. 1st ed. Switzerland: Springer International; 2017.

26. Mehta SR, Kumar S, Sing SP. Bio-terrorism—what should physicians know? Association of Physicians of India: medicine update. 2008;01(01):407–20. http://apiindia.org/wp-content/uploads/pdf/medicine_update_2008/chapter_53.pdf

27. Begier EM, Asiki G, Anywaine Z, Yockey B, Schriefer ME, Aleti P, et al. Pneumonic plague cluster, Uganda, 2004. Emerg Infect Dis. 2006;12:460–7. PubMed https://doi.org/10.3201/eid1203.051051

28. Brygoo ER, Gonon M. An epidemic of pneumonic plague in northeast Madagascar [in French]. Bull Soc Pathol Exot. 1958;51:47–60. **PMID: 13536775**

29. Burmeister RW, Tigertt WD, Overholt EL. Laboratory-acquired pneumonic plague. Report of a case and review of previous cases. Ann Intern Med. 1962;56:789–800. PubMed https://doi.org/10.7326/0003-4819-56-5-789

30. Champetier de Ribes G, Rasoamanana B, Randriambelosoa J, Rakoto LJ, Rabescn D, Chanteau S. The plague in Madagascar: epidemiologic data from 1989 to 1995 and the national control program [in French]. Sante. 1997;7:53–60. PubMed

31. Cohen RJ, Stockard JL. Pneumonic plague in an untreated plague-vaccinated individual. JAMA. 1967;202:365–6. PubMed https://doi.org/10.1001/jama.1967.03130170165036

32. Cramer C, Christensen B. Pneumonic plague in a 15-year-old Utah girl. J Emerg Nurs. 1995;21:491–3. PubMed https://doi.org/10.1016/S0099-1767(05)80257-0

33. Dawa W, Pan WJ, Gu XY, Zhang SQ, Dawa C, Yi X, et al. [Clinical features, diagnosis and treatment of 5 cases of primary pneumonic plague in Tibet in 2010] [in Chinese]. Zhonghua Jie He He Hu Xi Za Zhi. 2011;34:404–8. PubMed

34. Decker, J. Unusual manifestation of pneumonic plague. Cent Afr J Med. 1976;22:22–3.

35. Forrester JD, Apangu T, Griffith K, Acayo S, Yockey B, Kaggwa J, et al. Patterns of human plague in Uganda, 2008–2016. Emerg Infect Dis. 2017;23:1517–21. PubMed https://doi.org/10.3201/eid2309.170789

36. Gage KL, Dennis DT, Orloski KA, Ettestad P, Brown TL, Reynolds PJ, et al. Cases of cat-associated human plague in the Western US, 1977-1998. Clin Infect Dis. 2000;30:893–900. PubMed https://doi.org/10.1086/313804

37. Ge P, Xi J, Ding J, Jin F, Zhang H, Guo L, et al. Primary case of human pneumonic plague occurring in a Himalayan marmot natural focus area Gansu Province, China. Int J Infect Dis. 2015;33:67–70. PubMed https://doi.org/10.1016/j.ijid.2014.12.044

38. Ghosh PK. An outbreak of plague in an epidemic form treated with streptomycin and sulfadiazine. Ind Med Gaz. 1950;85:441–5. PubMed

39. Guillier G. Considération sur deux cas de peste pulmonaire traités par la streptomycine. Bull Soc Pathol Exot. 1953;46:622.

40. Gupta ML, Sharma A. Pneumonic plague, northern India, 2002. Emerg Infect Dis. 2007;13:664–6. PubMed https://doi.org/10.3201/eid1304.051105

41. Huang CH, Huang CY, Chu LW, Huang TF. Pneumonic plague; a report of recovery in a proved case and a note on sulfadiazine prophylaxis. Am J Trop Med Hyg. 1948;28:361–71. PubMed https://doi.org/10.4269/ajtmh.1948.s1-28.361

42. Joshi K, Thakur JS, Kumar R, Singh AJ, Ray P, Jain S, et al. Epidemiological features of pneumonic plague outbreak in Himachal Pradesh, India. Trans R Soc Trop Med Hyg. 2009;103:455–60. PubMed https://doi.org/10.1016/j.trstmh.2008.11.026

43. Kamugisha ML, Gesase S, Minja D, Mgema S, Mlwilo TD, Mayala BK. Pattern and spatial distribution of plague in Lushoto, north-eastern Tanzania. Tanzan Health Res Bull. 2007;9:12–8. PubMed https://doi.org/10.4314/thrb.v9i1.14286

44. Lewin W, Becker BJP, Horwitz B. Two cases of pneumonic plague: recovery of one case treated with streptomycin. S Afr Med J. 1948;22:699–703.

45. Li YF, Li DB, Shao HS, Li HJ, Han YD. Plague in China 2014-All sporadic case report of pneumonic plague. BMC Infect Dis. 2016;16:85. PubMed https://doi.org/10.1186/s12879-016-1403-8

46. Luo H, Dong X, Li F, Xie X, Song Z, Shao Z, et al. A cluster of primary pneumonic plague transmitted in a truck cab in a new enzootic focus in China. Am J Trop Med Hyg. 2013;88:923–8. PubMed https://doi.org/10.4269/ajtmh.12-0163

47. Madon MB, Hitchcock JC, Davis RM, Myers CM, Smith CR, Fritz CL, et al. An overview of plague in the United States and a report of investigations of two human cases in Kern county, California, 1995. J Vector Ecol. 1997;22:77–82. PubMed

48. McClean KL. An outbreak of plague in northwestern province, Zambia. Clin Infect Dis. 1995;21:650–2. PubMed https://doi.org/10.1093/clinids/21.3.650

49. McCrumb FR Jr, Mercier S, Robic J, Bouillat M, Smadel JE, Woodward TE, et al. Chloramphenicol and terramycin in the treatment of pneumonic plague. Am J Med. 1953;14:284–93. PubMed https://doi.org/10.1016/0002-9343(53)90040-0

50. Mercier S, Mac Crumb FR. [First cures of cases of pneumonic plague treated with terramycin]. Med Trop (Mars). 1952;12:698–706. PubMed

51. Mercier M. The first attempt of treatment of plague with chloromycetin [in undetermined language]. Bull Soc Pathol Exot. 1952;45:402–8.

52. Centers for Disease Control and Prevention (CDC). Bubonic and pneumonic plague—Uganda, 2006. MMWR Morb Mortal Wkly Rep. 2009;58:778–81. PubMed

53. Centers for Disease Control (CDC). Pneumonic plague—Arizona, 1992. MMWR Morb Mortal Wkly Rep. 1992;41:737–9. PubMed

54. Ramasindrazana B, Andrianaivoarimanana V, Rakotondramanga JM, Birdsell DN, Ratsitorahina M, Rajerison M. Pneumonic plague transmission, Moramanga, Madagascar, 2015. Emerg Infect Dis. 2017;23:521–4. PubMed https://doi.org/10.3201/eid2303.161406

55. Richard V, Riehm JM, Herindrainy P, Soanandrasana R, Ratsitoharina M, Rakotomanana F, et al. Pneumonic plague outbreak, Northern Madagascar, 2011. Emerg Infect Dis. 2015;21:8–15. PubMed https://doi.org/10.3201/eid2101.131828

56. Roux AH, Mercier C. Sur cinq cas de peste pulmonaire primitive dont trois suivis de guérison, observés à l'hôpital civil d'Oran. Bull Soc Pathol Exot. 1946;39:173–8.

57. Runfola JK, House J, Miller L, Colton L, Hite D, Hawley A, et al.; Centers for Disease Control and Prevention (CDC). Outbreak of human pneumonic plague with dog-to-human and possible human-to-human transmission—Colorado, June–July 2014. MMWR Morb Mortal Wkly Rep. 2015;64:429–34. PubMed

58. Seal SC, Prasad G. Further notes on the incidence of pneumonic plague cases in Gaya (Bihar). Ind Med Gaz. 1949;84:408–13. PubMed

59. Seal SC. Pneumonic plague cases in Calcutta and Gaya. Ind Med Gaz. 1949;84:162–70. PubMed

60. Tieh TH, Landauer E, Miyaga F, Kobayashi G, Okayasu G. Primary pneumonic plague in Mukden, 1946, and report of 39 cases with three recoveries. J Infect Dis. 1948;82:52–8. PubMed https://doi.org/10.1093/infdis/82.1.52

61. Trong P, Nhu TQ, Marshall JD Jr. A mixed pneumonic bubonic plague outbreak in Vietnam. Mil Med. 1967;132:93–7. PubMed https://doi.org/10.1093/milmed/132.2.93

62. Wagle PM, Bedarkar MK. Pneumonic plague and its treatment. Ind Med Gaz. 1948;83:406–9. PubMed

63. Wang H, Cui Y, Wang Z, Wang X, Guo Z, Yan Y, et al. A dog-associated primary pneumonic plague in Qinghai Province, China. Clin Infect Dis. 2011;52:185–90. PubMed https://doi.org/10.1093/cid/ciq107

64. Werner SB, Weidmer CE, Nelson BC, Nygaard GS, Goethals RM, Poland JD, et al. Primary plague pneumonia contracted from a domestic cat at South Lake Tahoe, Calif. JAMA. 1984;251:929–31.

65. Wong D, Wild MA, Walburger MA, Higgins CL, Callahan M, Czarnecki LA, et al. Primary pneumonic plague contracted from a mountain lion carcass. Clin Infect Dis. 2009;49:e33–8. PubMed https://doi.org/10.1086/600818

66. Wu KM, Yang YH, Wang YZ, Wang X, Qi ZZ, Wang ZY. Epidemiological analysis of plague in Qinghai Province between 2000 and 2009. Zhonghua Liu Xing Bing Xue Za Zhi. 2011;30:437–40.

67. Zhu J. [Analysis of human plague episodes in Qinghai from 1958 to 1991]. Zhonghua Liu Xing Bing Xue Za Zhi. 1993;14:227–30. PubMed

Appendix Table. Reports of pneumonic plague, 1946–2017*

Author/year	First year cases described	Country	Total		Antimicrobial drug use unknown		Treated patients		Untreated patients	
			Deaths	Patients	No. patient deaths	No, patients with unknown use	No. deaths	No. treated	No. deaths	No. patients
Begier (2006) (*27*)	2004	Uganda	1	2			0	1	1	1
Brygoo 1958) (*28*)	1957	Madagascar	35	41			0	6	35	35
Burmeister (1962) (*29*)	1959	USA	0	1			0	1		
de Ribes 1997) (*30*)	1989	Madagascar	52	91	52	91				
Cohen (1967) (*31*)	1966	Vietnam	0	1			0	1		
Cramer (1995) (*32*)	1995	USA	0	1	0	1				
Dawa (2011) (*33*)	2010	Tibet	0	5			0	5		
Decker (1976) (*34*)	1975	Zimbabwe	0	1			0	1		
Donaires (2010) (*20*)	2010	Peru	2	4			2	4		
Forrester (2017) (*35*)	2008	Uganda	8	18	8	18				
Gage (2000) (*36*)	1977	USA	2	5	2	5				
Ge (2015) (*37*)	2014	China	1	1			1	1		
Ghosh (1950) (*38*)	1950	India	0	19			0	19		
Guillier (1953) (*39*)	1948	Madagascar	0	2			0	2		
Gupta (2007) (*40*)	2002	India	4	16	4	16				
Huang (1948) (*41*)	1947	China	0	1			0	1		
Joshi (2009) (*42*)	2002	India	5	30	5	30				
Kamugisha (2007) (*43*)	1986	Tanzania	121	427	121	427				
Lewin (1948) (*44*)	1947	South Africa	1	2			1	2		
Li (2016) (*45*)	2014	China	3	3			1	1	2	2
Luo (2013) (*46*)	2005	China	2	5			1	3	1	2
Madon (1997) (*47*)	1995	USA	1	1			1	1		
McClean (1995) (*48*)	1993	Zambia	2	3			1	2	1	1
McCrumb (1953) (*49*)	1953	Madagascar	2	13			2	13		
Mercier (1952) (*50*)	1952	Madagascar	4	8			0	4	4	4
Mercier (1952) (*51*)	1951	Madagascar	0	1			0	1		
Mwengee (2006) (*9*)	2002	Tanzania	1	1			1	1		
Ogen-Odoi (2009) (*52*)	2006	Uganda	11	12	11	12				
Opulski (1992) (*53*)	1992	USA	1	1			1	1		
Ramasindrazana (2017) (*54*)	2015	Madagascar	6	14						
Ratsitorahina (2000) (*1*)	1997	Madagascar	7	17			1	9	7	7
Richard (2015) (*55*)	2011	Madagascar	15	20			0	5	15	15
Roux (1946) (*56*)	1946	Algeria	2	4			2	4		
Runfola (2015) (*57*)	2014	USA	0	3			0	3		
Seal (1949) (*58*)	1948	India	11	12	11	12				
Seal (1949) (*59*)	1948	India	13	14			0	1	13	13
Tieh (1948) (*60*)	1946	China	36	39					36	39
Trong (1967) (*61*)	1965	Vietnam	18	43	18	43				
Wagle (1948) (*62*)	1948	India	0	3			0	3		
Wang (2011) (*63*)	2009	China	3	12			2	11	1	1
Werner (1984) (*64*)	1980	USA	1	1			1	1		
Wong (2009) (*65*)	2007	USA	1	1					1	1
Wu (2011) (*66*)	2000	China	9	26	9	26				
Zhu (1993) (*67*)	1958	China	98	182	98	182				

*Blank cells indicate data not available.

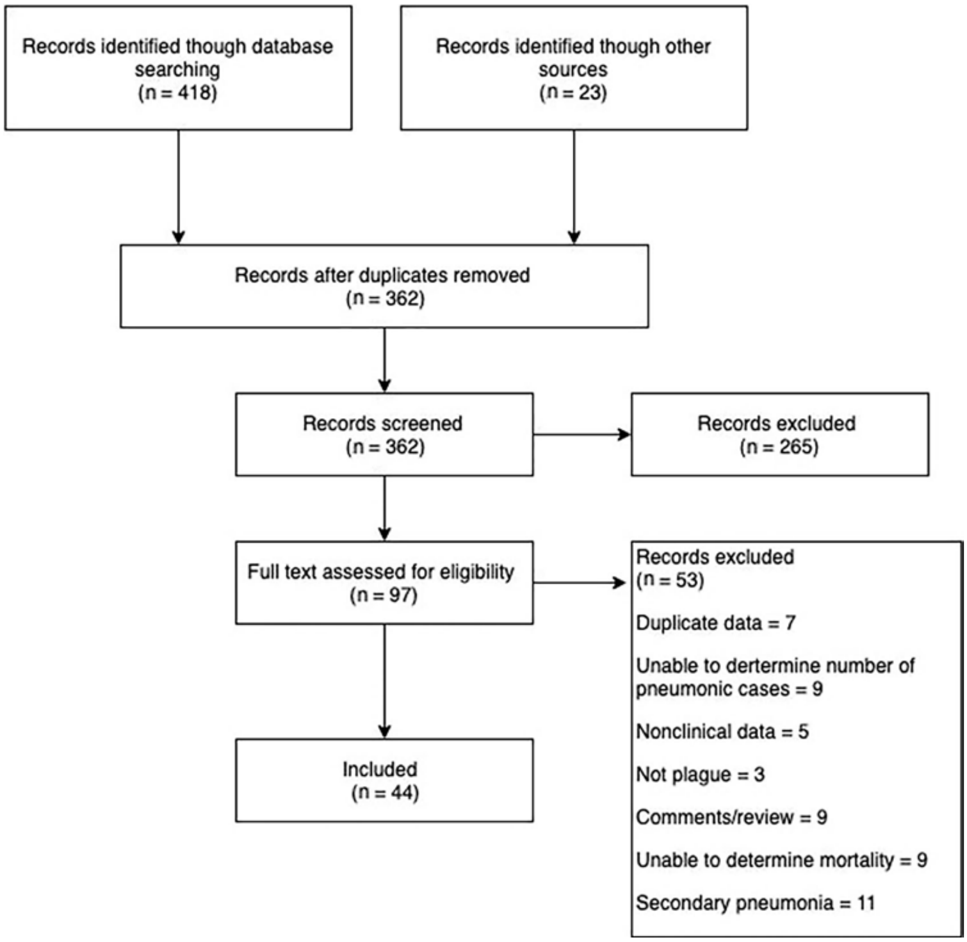

Appendix Figure 1. Overview of 362 reports of pneumonic plague, 1946–2017.

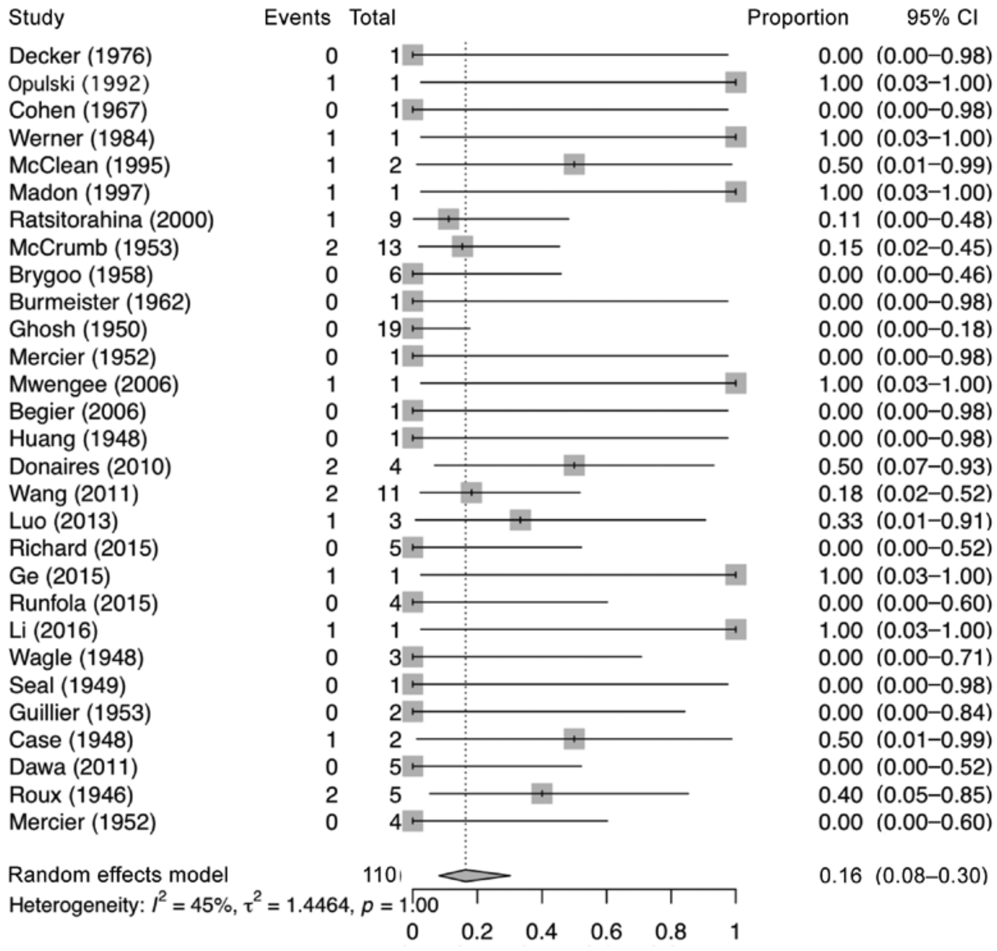

Appendix Figure 2. Forrest plot for death of all patients receiving antibiotics treatment for pneumonic plague in various countries, 1946–2015.

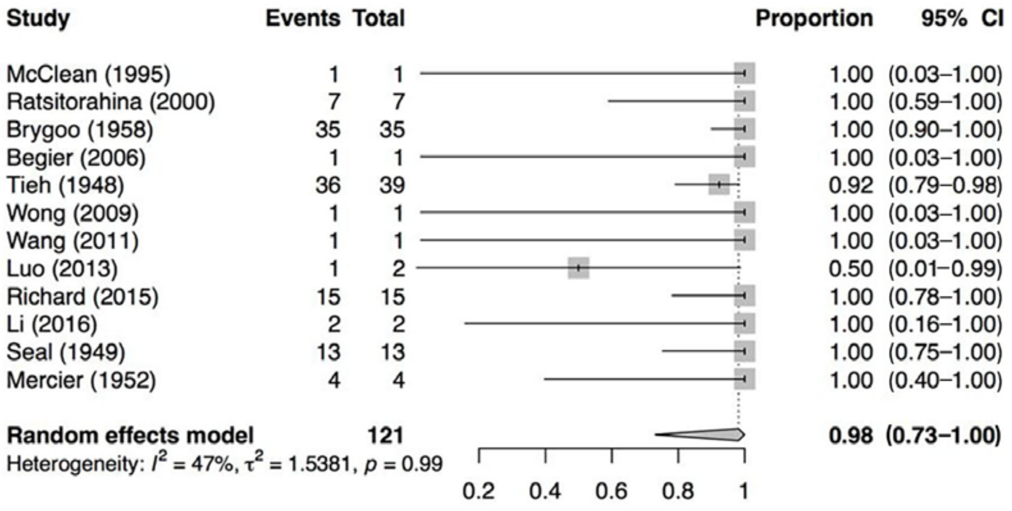

Appendix Figure 3. Forest plot for death of all patients with untreated pneumonic plague in various countries, 1967–2017.

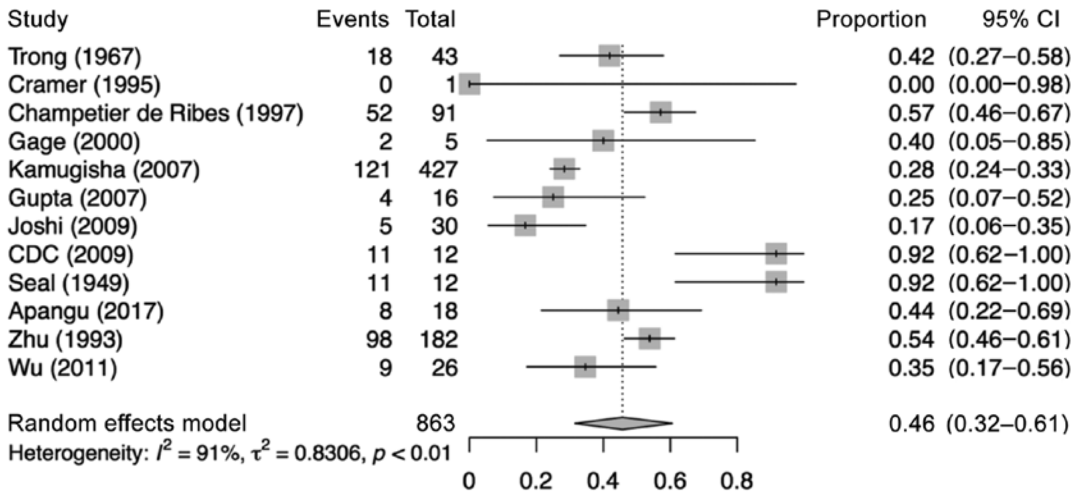

Appendix Figure 4. Forest plot for pneumonic plague patients where antibiotic status was unknown, 1949–2016. Refer to Appendix Table for full citations.
